# Using systems archetypes to understand system behaviour and identify leverage points for change in local obesity prevention in The Netherlands

**DOI:** 10.1093/heapro/daag056

**Published:** 2026-05-19

**Authors:** Jillian O’Mara, Loes Crielaard, Luc Hagenaars, Karien Stronks, Wilma Waterlander

**Affiliations:** Department of Public and Occupational Health, Amsterdam UMC Location University of Amsterdam, Meibergdreef 9, Amsterdam, 1105 AZ, The Netherlands; Health Behaviours and Chronic Diseases, Amsterdam Public Health Research Institute, Amsterdam, 1105 AZ, The Netherlands; Department of Public and Occupational Health, Amsterdam UMC Location University of Amsterdam, Meibergdreef 9, Amsterdam, 1105 AZ, The Netherlands; Health Behaviours and Chronic Diseases, Amsterdam Public Health Research Institute, Amsterdam, 1105 AZ, The Netherlands; Department of Public and Occupational Health, Amsterdam UMC Location University of Amsterdam, Meibergdreef 9, Amsterdam, 1105 AZ, The Netherlands; Health Behaviours and Chronic Diseases, Amsterdam Public Health Research Institute, Amsterdam, 1105 AZ, The Netherlands; Department of Public and Occupational Health, Amsterdam UMC Location University of Amsterdam, Meibergdreef 9, Amsterdam, 1105 AZ, The Netherlands; Health Behaviours and Chronic Diseases, Amsterdam Public Health Research Institute, Amsterdam, 1105 AZ, The Netherlands; Department of Public and Occupational Health, Amsterdam UMC Location University of Amsterdam, Meibergdreef 9, Amsterdam, 1105 AZ, The Netherlands; Health Behaviours and Chronic Diseases, Amsterdam Public Health Research Institute, Amsterdam, 1105 AZ, The Netherlands

**Keywords:** systems archetypes, system dynamics, participatory system dynamics, systems thinking, causal loop diagram, complexity, obesity prevention

## Abstract

Participatory system dynamics (SD) approaches view public health problems as the result of a complex system of interactions. However, most public health research makes a direct leap from system mapping—often through causal loop diagrams (CLDs)—to identifying actions for change, without trying to understand the behaviour of the system as a whole and how actions could intervene. This is possibly because robust methods are lacking. This study aimed to explore whether ‘systems archetypes’ can bridge this gap to (1) better understand system behaviour, (2) identify leverage points (LPs) for change deeper in the system, and (3) provide a more structured and traceable analysis. We developed a novel approach using 11 systems archetypes for a *post hoc* analysis of the LIKE project—a participatory SD project on childhood obesity prevention in Amsterdam, the Netherlands. For each LIKE mechanism, we compiled a complete archetype profile, including the storyline, CLD, and behaviour over time graph, for which two were cross-checked with empirical data over time. We identified six systems archetypes. The most common was ‘fixes that fail’, in which a ‘fix’ applied to a problem creates unintended consequences that reinforce the problem. This can lead to ‘shifting the burden’ in which resources are siphoned from addressing the root cause of the problem. Compared with the original analysis, we identified LPs deeper in the system and found that systems archetypes structured the process, suggesting that systems archetypes can effectively help public health researchers hypothesize how to change system behaviour.

Contribution to Health Promotion

- Many public health problems such as obesity and socioeconomic health inequalities are complex and require complex approaches.
- Public health researchers, policymakers, and practitioners are increasingly turning to (participatory) system dynamics to help understand the system driving health problems and look for ways to change it.
- Systems archetypes are relatively new in public health and have the potential to help us hypothesize about system behaviour and prescribe better change strategies which could promote better public health outcomes.
- Systems archetypes should be added to the system dynamics toolbox in public health for researchers, policymakers and practitioners.

## Background

Public health problems, such as obesity and socioeconomic health inequalities, are increasingly conceptualized as the product of a complex system of factors spanning diverse domains that, through their interactions, behave in unpredictable ways (Carey *et al*. 2015, Rutter *et al*. 2017, Greenhalgh and Papoutsi 2018, Rod *et al*. 2023, Stronks *et al*. 2024). Methods from the field of system dynamics (SD) have gained traction to understand this complexity (Stronks *et al*. 2024, Crielaard *et al*. 2025). These include both quantitative methods (e.g. SD models that simulate system behaviour) and qualitative methods [e.g. causal loop diagrams (CLDs) that visualize system structure] (Cilenti *et al*. 2019, Crielaard *et al*. 2025). SD approaches, which focus on anticipating how an intervention may interact with the broader system, are a response to the limitations of traditional ‘linear’ health interventions in addressing complex public health problems. Such interventions tend to assume straightforward cause‒effect relationships, which for complex problems could result in a lack of long-term efficacy, often resulting from unintended consequences for which the linear intervention fails to account (Rutter *et al*. 2017, Gadsby and Wilding 2024).

Specifically, participatory SD approaches have gained momentum in public health, which have the additional characteristic that stakeholders are engaged throughout the process of modelling and intervening in a system. For example, three projects that were identified in a systematic review for having applied a (participatory) SD approach in obesity prevention from inception to completion, WHOSTOPS (Allender *et al*. 2016), RESPOND (Whelan *et al*. 2020), and LIKE (Waterlander *et al*. 2020), as well as the more recent Generation Healthy Kids Study (Ryom *et al*. 2025), used (forms of) ‘group model building’ (GMB) to collaboratively establish an understanding of a system's feedback structures by drawing CLDs (Luna Pinzon *et al*. 2022). These projects then relied on group discussions to hypothesize where system change might be most effectively leveraged (Li *et al*. 2023), often on the basis of frameworks that distinguish different levels of change, such as Meadows’ 12 Places to Intervene in a System (Meadows 2008), the Intervention Level Framework (ILF) (Johnston *et al*. 2014), or the Action Scales Model (Nobles *et al*. 2022). The ILF, for example, defines five levels on which one might intervene in a system ranging from the shallow level ‘structural elements’ to the deep level ‘system paradigm’ (see Table 3) (Johnston *et al*. 2014). Intervening at deeper levels is theorized to be more effective in leveraging system change because this means targeting the fundamental, underlying core beliefs and paradigms. An example of a system paradigm is the social norm that unhealthy behaviour is ‘cool’. However, the deep levels are more difficult to target and change. Intervening at shallower levels is theorized to have less effect on the system’s outcome but is easier to address, such as changing structural elements in the system (Johnston *et al*. 2014, Stronks *et al*. 2024). An example of an intervention at the level of structural elements is introducing health ambassadors that promote healthy lifestyle to local residents. Using such frameworks aims to facilitate the targeting of leverage points (LPs) at the deeper levels to effectively leverage change in the system.

These approaches share the strength that qualitative CLDs helped visualize the complexity of the problem at hand and identify LPs and actions for change at different system levels, but they face two limitations. First, the frameworks do not explicitly facilitate hypothesizing about the system’s behaviour based on the system’s structure, as represented by the feedback loops in the CLD (Gerritsen *et al*. 2019, Crielaard *et al*. 2024). This leaves it open to researcher interpretation, which limits reproducibility. It also often limits the identification of LPs to specific sections of the system, such as subsystems or feedback loops, making it difficult to intervene on the behaviour of the system as a whole. Second, although these frameworks support the identification of LPs intended to disrupt underlying mechanisms, they do not make explicit how proposed actions may alter current behaviour. This makes it challenging to reflect on their potential effectiveness (Uleman *et al*. 2021). Accordingly, the initial aim of using SD to identify LPs and develop actions that target deeper levels of the system often remains elusive. For example, in the LIKE project, only three of the 14 actions aimed at leveraging system change were at the paradigm level (Luna Pinzon *et al*. 2024). These actions included, i.e., attempts to change social norms around healthy behaviour by engaging with peer role models and creating a role model network.

Besides the aforementioned frameworks to identify LPs based on a CLD, to our knowledge, there is no tool consistently being used in qualitative systems approaches in public health to bridge the gap between system structure and system behaviour and ultimately LPs for change. One method from the SD literature that might suit this goal is systems archetypes. Systems archetypes were developed through the synthesis of work from SD pioneers, including Forrester, Meadows, Senge, and Kim (Kim and Anderson 1998, Kim 2000a, 2000b, 2000c, Senge 2006, Meadows 2008). Meadows defines systems archetypes as, ‘Common system structures that produce characteristic patterns of [problematic] behaviour’ (Meadows 2008), such as ‘success to the successful’ in which success for one person, organization, or group continues to grow while undermining the success of the other; or ‘tragedy of the commons’ in which vying for a common resource ultimately depletes that resource. Kim (2000a) defines archetypes more operationally, stating that ‘[Archetypes] are powerful tools for diagnosing problems and identifying high-leverage interventions that will create fundamental change’, thus positing archetypes as a method for leveraging change ‘deeper’ in the system. Meadows and Kim both emphasize the use of archetypes as tools to structure the process from (the visualization of) system structure to leveraging change, connecting structure to hypotheses about behaviour and subsequently prescribing change strategies for actioning change in the system.

Systems archetypes are relatively new in SD approaches in public health, compared with e.g. the use of CLDs. To illustrate, while a recent scoping review on CLDs describing food systems in relation to diet and obesity identified 40 CLDs in this niche area alone (Stankov *et al*. 2025), in our forthcoming review on systems archetypes, we found only 29 applications of systems archetypes across the domains of public health, healthcare policy, and occupational health—covering topics ranging from mental health to dialysis services and occupational safety—highlighting that the use of systems archetypes as a tool from the SD toolbox remains relatively rare. According to Meadows and Kim, systems archetypes have the potential to guide the steps between understanding system structure and theorizing about system behaviour and LPs. Therefore, systems archetypes may be an important addition to the currently available frameworks and methods to address complex public health problems. In order to explore the potential unique advantage of systems archetypes as an additional method in SD approaches in public health, this study aims to analyse if, in comparison with the previous analysis (see Box 1), the use of systems archetypes:

1. Helps to hypothesize about the behaviour of the system.
2. Allows for the identification of LPs and the development of actions at deeper levels (as defined by the ILF) of the system.
3. Provides a more structured and traceable method to link system structure to system behaviour and ultimately to LPs for change.

Box 1 Case study—LIKE project and selected mechanisms for analysis. An example of a project that applied a participatory SD approach is the Lifestyle Innovations based on youth’s Knowledge and Experience (LIKE) project, which aimed to reduce childhood overweight and obesity in Amsterdam, the Netherlands, by promoting healthier living environments (Waterlander *et al*. 2020). The LIKE project involved stakeholders from inception to conclusion, including the research team, the city of Amsterdam, the municipal health services, the funding partners, schools, and youth living in Amsterdam East and their parents. The project employed a variety of SD tools and methods. These included an in-depth, mixed-methods, multistakeholder needs assessment that aimed to capture the underlying system dynamics driving obesity-related behaviours in youth in Amsterdam and the subsequent development of a causal loop diagram (CLD) that sought to map the existing system (Waterlander *et al.* 2021, Luna Pinzon *et al.* 2022, 2023, Emke *et al.* 2025).

The central CLD that was developed during LIKE to understand the system and develop leverage points for change identified 121 factors and 31 feedback loops and was divided into six subsystems: (1) interaction between adolescents and the food environment, (2) interaction between adolescents and the physical activity environment, (3) interaction between adolescents and the online environment, (4) interaction between adolescents, parenting and the wider socioeconomic environment, (5) interaction between healthcare professionals and adolescents with obesity and their parents, and (6) transition from childhood to adolescence (Luna Pinzon *et al*. 2023). The CLD was used to identify the underlying mechanisms based on group discussions in which the question was asked: ‘Taking into account the needs assessment results, what are the most important mechanisms contributing to unhealthy lifestyles among adolescents aged 10–14?’ For eight identified mechanisms, LPs were specified, and intervention actions were created, guided by the Intervention Level Framework. These mechanisms, which were the starting point for the analysis in this paper, can be found in Table 1. A total of 14 actions based on nine LPs were implemented. A full description of this process can be found here (Luna Pinzon *et al*. 2024).

**Table 1:** Results of the analysis using systems archetypes to interpret the mechanisms as formulated in the LIKE project..

**1**	**Archetype description (theory from system dynamics literature)**	**Archetype: fixes that fail** **General description:** The ‘fixes that fail’ archetype shows a pattern in which a fix is applied to a problem. While the fix may temporarily relieve the problem symptoms, it produces unintended consequences, which over time reinforce the problem and the fix fails (Kim 2000a).		
	**Mechanism as formulated in LIKE (** Luna Pinzon *et al*. 2024 **)**	**Match between obesity healthcare services and the needs of adolescents with obesity and their parents (M7 in LIKE)** The working methods, organization, and competences of healthcare professionals do not sufficiently fit the needs and possibilities of the multiethnic target group in Amsterdam East.		
	**Newly formulated LIKE archetype (LA1)**	**Storyline** The health professionals have the good intention of helping families with children with obesity. However, the working methods, competences (and therefore programmes, solutions etc.) offered to the multiethnic target group do not fit their needs and therefore fail. The unintended consequence is that the families have a negative experience related to obesity care, making it even more difficult to support them. The children who needs care do not receive it, making it less likely that their situation improves, and other children in the same community may be influenced to not seek care, possibly leading to higher prevalence of children with obesity.	**Causal loop diagram** 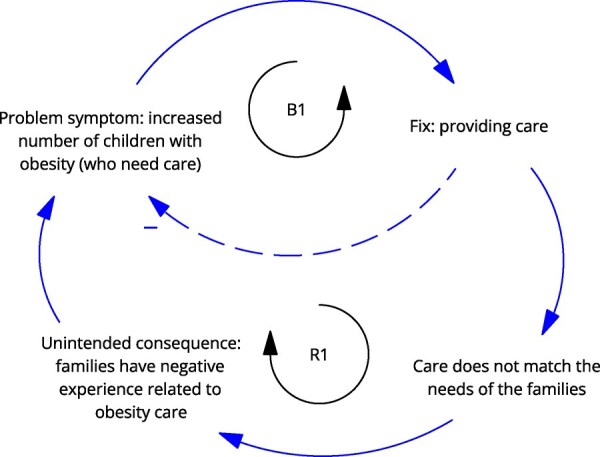	**Graph over time** 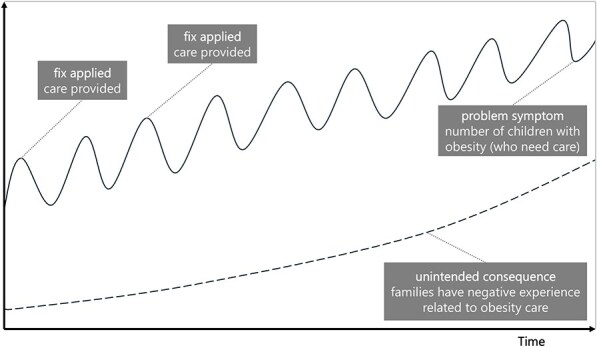
**2**	**Archetype Description (theory)**	**Archetype: fixes that fail—shifting the burden** **General description:** A ‘fixes that fail’ archetype can be paired with a ‘shifting the burden’ archetype over time. This happens when an additional side-effect adds a reinforcing loop that takes attention/resources away from addressing the fundamental solution (Kim 2000a).		
	**Mechanism as formulated in LIKE (** Luna Pinzon *et al*. 2024 **)**	**The use of public outdoor spaces for physical activity by adolescents (M2 in LIKE)** In highly populated cities such as Amsterdam, the high demand for housing and business has resulted in public outdoor spaces designed for active play and sports being built on the outskirts of neighbourhoods. Larger distances to such spaces are a barrier for adolescents. Furthermore, public outdoor spaces are generally unattractive for adolescents as they are often designed for younger children, or adults, or for one specific activity, for example, basketball or soccer. Furthermore, adolescents are not engaged in the decision-making/design/organization of public outdoor spaces, resulting in these spaces not matching their wishes and/or needs.		
	**Newly formed LIKE archetype (LA2)**	**Storyline** The problem is that adolescents are not physically active (enough), leading to the problem symptom ‘the need to do ‘something’’ about adolescent inactivity in urban areas. The fix is building physical activity locations at the edge of the city. That fails because it is known that an important aspect of physical activity is daily practice, which comes from active transport, playing outside, etc., rather than traveling to a different neighbourhood for access to physical activity spaces (fix that fails). The side-effect is that the amenities built at the outskirts are not being used, leading to the impression that there is no need/no interest, which leads to even less investment in these kinds of amenities (in places in which they would be used) and reducing the attention and resources for the fundamental solution of creating appropriate physical activity (close to home) in urban areas (shifting the burden).	**Causal loop diagram** 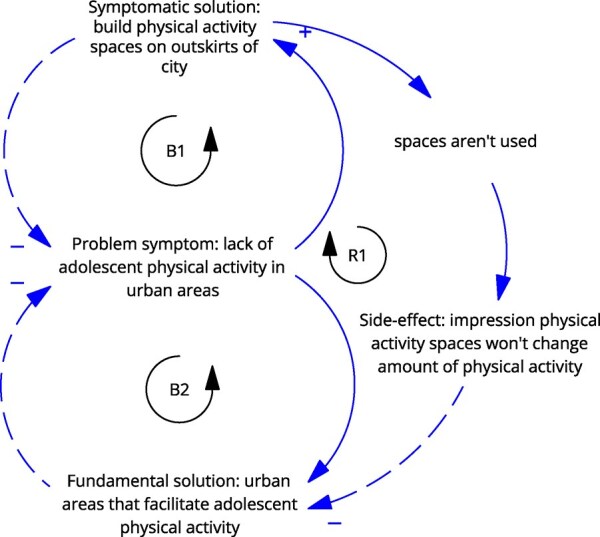	**Graph over time** 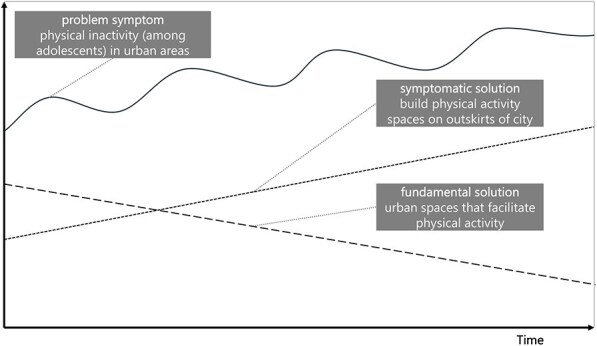
**3**	**Archetype Description (theory)**	**Archetype: Fixes that fail—Shifting the burden** A ‘fixes that fail’ archetype can be paired with a ‘shifting the burden’ archetype over time. This happens when an additional side-effect adds a reinforcing loop that takes attention/resources away from addressing the fundamental solution (Kim 2000a).		
	**Mechanism as formulated in LIKE (** Luna Pinzon *et al*. 2024 **)**	**Livelihood security and poverty (M4 in LIKE)** When families live in relative poverty, the problems and stress they experience may occupy parents’ headspace. As a result, they often pay less attention to stimulating healthy behaviours in their children.		
	**Newly formed LIKE archetype (LA3)**	**Storyline** The problem is that people with lower socioeconomic status have more health problems. The ‘fix’ is to offer ‘effective’ health programmes. The unintended consequence is that people with lower socioeconomic status become aware they ‘are not doing it right’, while they still do not have the headspace to truly focus on this aspect of their lives, which adds to the already high stress levels and serves to exacerbate the original problem (fix that fails). The side-effect is that health is framed as an individual problem and prevention budgets are spent on individual health programmes. This takes away from the fundamental solution of addressing root causes of poverty and stress (shifting the burden).	**Causal loop diagram** 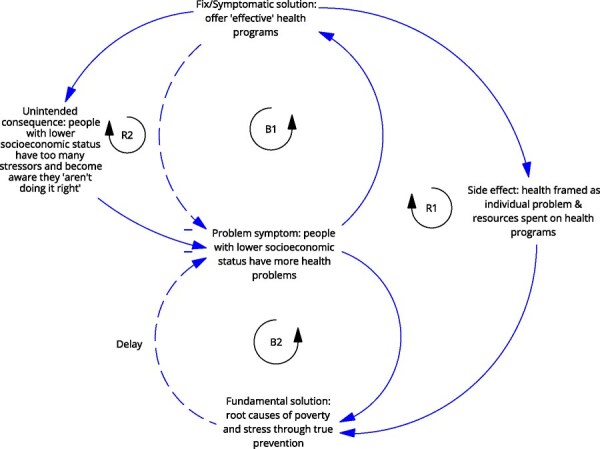	**Graph over time** 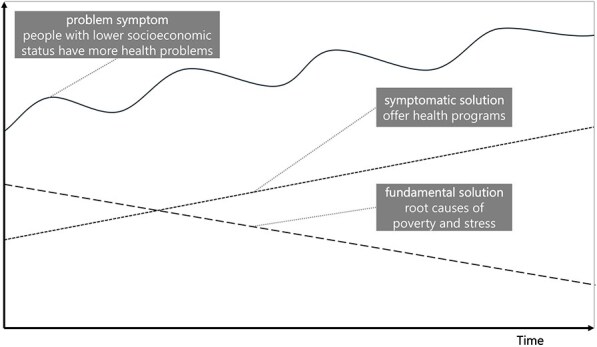
**4**	**Archetype Description (theory)**	**Archetype: Fixes that fail—Accidental adversaries** **General description:** A ‘fixes that fail’ can also result in an ‘accidental adversaries’ over time when the failure to fix the problem results in distrust between two parties (Kemeny 1994).		
	**Mechanism as formulated in LIKE (** Luna Pinzon *et al*. 2024 **)**	**Match between local health promotion activities and parents’ needs (M6 in LIKE)** In the LIKE focus area, there are many health promotion activities organized for parents. However, there is a mismatch between the content and type of such activities and the needs and wishes of parents. Because of this, professionals organizing these activities and parents attending such activities have problems in communicating and understanding each other. This in turn can lead to demotivation, misunderstanding and uncertainty among both parents and professionals organizing the health promotion activities, thereby forming a barrier to the promotion of a healthier lifestyle.		
	**Newly formed LIKE archetype (LA4)**	**Storyline** The problem is unhealthy lifestyle of youth in Amsterdam. The applied ‘fix’ is health promotion activities run by professionals. The health promotion services have good intentions in providing care and ensuring the healthy growth and development for children. However, over time, it becomes apparent that the offered ‘fixes’ do not correspond with the needs and wishes of the parents, so the fix fails. Over a longer period of time, the parents and the health professionals become ‘accidental adversaries’. They no longer understand one another—the parents feel their needs and wishes are not being addressed and the health professionals feel ‘betrayed’ by the people they were trying to help.	**Causal loop diagram** 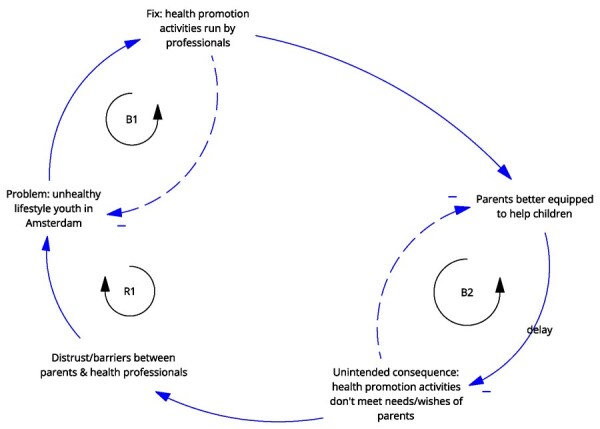	**Graph over time** 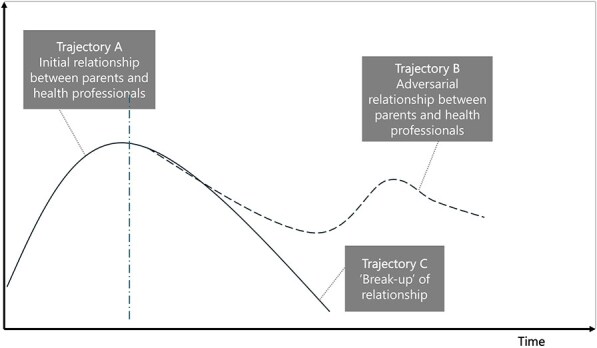
**5**	**Archetype Description (theory)**	**Archetype: Success to the successful** **General description:** The ‘success to the successful’ archetype occurs when individuals, groups, projects, etc., are competing for resources. One group receives more resources, which amplifies their success, leading to more success (reinforcing). This comes at the detriment of the other group, which receives fewer resources, less success, etc. (reinforcing) (Kim 2000a).		
	**Mechanism as formulated in LIKE (** Luna Pinzon *et al*. 2024 **)**	**Power dynamics in the current food system (M1 in LIKE)** In the current food subsystem, a power imbalance exists between large global food companies and smaller local companies. This imbalance results from the large profits made by global food companies on unhealthy food sales, which are more profitable than healthier options. These profits can in turn be further invested in, for example, marketing and lobbying, thereby further boosting the demand for unhealthy food.		
	**Newly formed LIKE archetype (LA5)**	**Storyline** The current food environment is dominated by unhealthy foods, which are produced and marketed to be as appealing as possible. Consumer food choice, which is the ‘resource’ is then allocated to unhealthy foods, triggering a reinforcing loop in which unhealthy foods gain more resources and more success, which can be defined by profits and proliferation (see Box 2). Through high demand, the cost of unhealthy food becomes even lower, leading to more supply, more resources for distribution, marketing, development, and even more efficiency in the supply chain, to even lower prices (and higher profits). This comes at the expense of (local) healthy food retailers, because small producers cannot compete (have lower profit margins) and experience even higher cost together with decreasing demand. This can lead to a decline in healthy food retailers. This example has been cross-checked with empirical data in Box 2.	**Causal loop diagram** 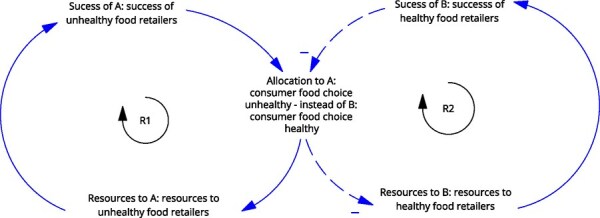	**Graph over time** 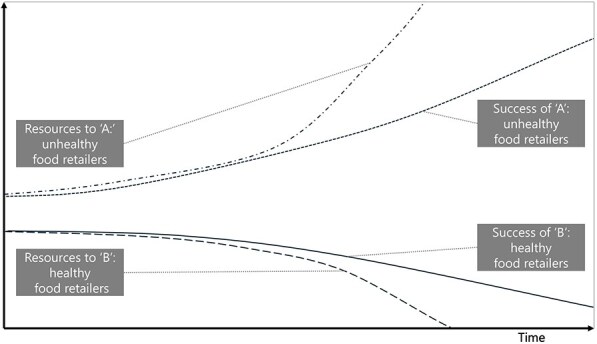
**6**	**Archetype Description (theory)**	**Archetype: Success to the successful** **General description:** The ‘success to the successful’ archetype occurs when individuals, groups, projects, etc., are competing for resources. One group receives more resources, which amplifies their success, leading to more success (reinforcing). This comes at the detriment of the other group, which receives fewer resources, less success, etc. (reinforcing) (Kim 2000a).		
	**Mechanism as formulated in LIKE (** Luna Pinzon *et al*. 2024 **)**	**Social norms influencing health behaviours in adolescents (M8 in LIKE)** Health behaviours are strongly influenced by social norms in a specific group and that behaviour in turn influences the social norm. During the transition from childhood to adolescence, adolescents typically desire to be part of and accepted by a group, thereby making them extra vulnerable to the influence of, for example, peers and friends. Adolescents usually exhibit unhealthy behaviours when hanging out with friends. The current social norm amongst adolescents is that unhealthy behaviour is cool.		
	**Newly formed LIKE archetype (LA6)**	**Storyline** Currently, in part due to advertising on/influence of social media, unhealthy behaviour has become the ‘norm’. This leads to an increase in attention for and adoption of unhealthy behaviour, which increases the ‘success’, meaning the occurrence, of unhealthy behaviour. Unhealthy behaviour becomes the more dominant norm. This is reinforced by the adolescent’s desire to ‘belong,’ which strengthens the reinforcing loop, and unhealthy behaviour as the norm. This norm dominates at the expense of the ‘healthy’ norm (as one norm becomes more dominant, the other becomes less dominant). The less dominant norm is seen less on social media, and over time declines in overall ‘success’, meaning it occurs less.	**Causal loop diagram** 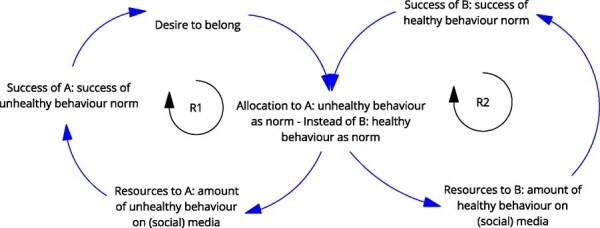	**Graph over time** 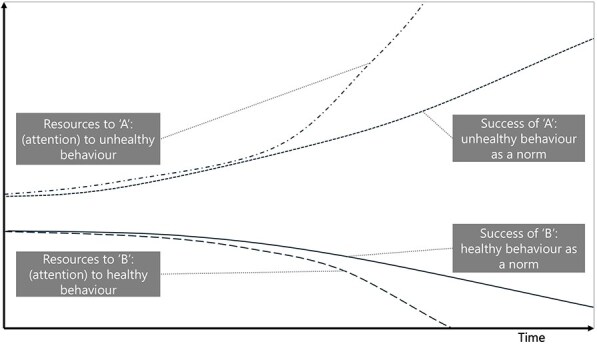
**7**	**Archetype Description (theory)**	**Archetype: Escalation** **General description:** The ‘escalation’ archetype occurs when two balancing loops of reaction and counter-reaction lead to an escalating situation (Kim 2000a).		
	**Mechanism as formulated in LIKE (** Luna Pinzon *et al*. 2024 **)**	**The role of parents during adolescence (M3 in LIKE)** Parents undergo a new role from a managerial to a more coaching role of their children in the transition from child to adolescent. Because of this, they may find it difficult to set, monitor and enforce rules regarding sleep, dietary behaviour, screen behaviour, and physical activity. Adolescents have difficulties making sensible choices regarding bedtime and screen use in the evenings and indicate that they need their parents to set rules		
	**Newly formed LIKE archetype (LA7)**	**Storyline** In the relationship (parent‒child/adolescent), party A (the parent) takes action against a (perceived) threat: too much screen use. This begins with a mild action, such as telling the adolescent not to be on their phone so much. Party B (the adolescent) feels an imbalance of power and has a counter-reaction such as getting angry, ignoring the parent, arguing, secretly using the phone. This reaction might be stronger than the initial parental action (possibly bolstered by puberty). The parents try to regain grip and react more heavily, by setting rules, which causes the adolescent to react even more strongly. The parents lack options, and the situation continues to escalate.	**Causal loop diagram** 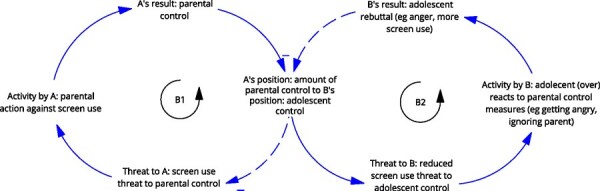	**Graph over time** 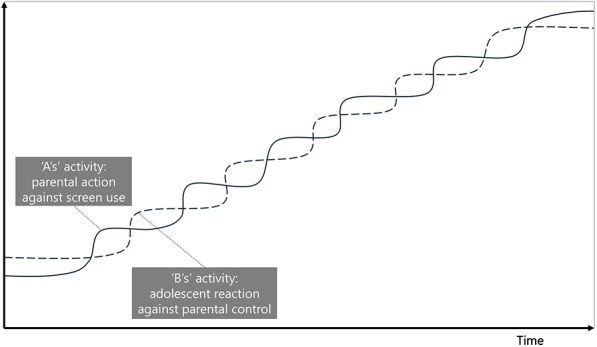
**8**	**Archetype Description (theory)**	**Archetype: Limits to success** **General description:** The ‘limits to success’ archetype occurs when initial growth is hindered by a constraint, leading to stalled growth and limited success (Kim 2000a).		
	**Mechanism as formulated in LIKE (** Luna Pinzon *et al*. 2024 **)**	**Connection between health ambassadors (volunteers), municipality, and community organizations (M5 in LIKE)** In the LIKE focus area, community health ambassadors, who are mostly parents, play an important role in stimulating local residents (such as fellow parents) towards developing healthier habits. However, the support that health ambassadors receive from the municipality and community organizations does not sufficiently match their wishes and expectations. This results in less and less health ambassadors committing to influencing local residents towards a healthier lifestyle		
	**Newly formed LIKE archetype (LA8)**	**Storyline** At first, the volunteer health ambassadors found success in helping (fellow parents) towards developing healthier habits. The number and capacity of volunteer ambassadors grew, and they started to have more and more responsibility. The limit (constraint) was the compensation and support from the local municipality and community organizations. The municipality and community organizations were unable to provide the ambassadors with support that matched their needs and expectations. This resulted in fewer health ambassadors, despite their efforts.	**Causal loop diagram** 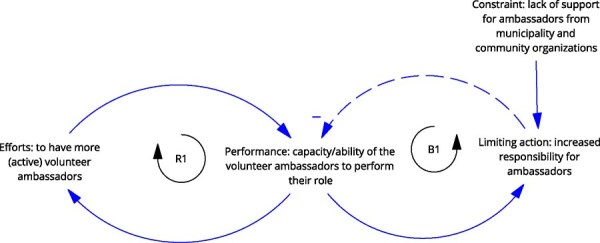	**Graph over time** 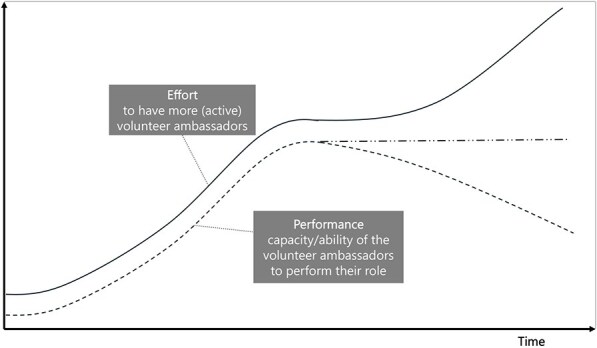

Causal loop diagram: solid line arrows indicate a positive relationship meaning the factors both increase or both decrease, dashed line arrows indicate an inverse relationship meaning when one factor increases, the other decreases, balancing and reinforcing loops are indicated by ‘B1’ ‘B2’ or ‘R1’ ‘R2’.

## Methods

### Design

We performed a qualitative *post hoc* analysis of the LIKE project, which is an appropriate case for exploring the added value of systems archetypes within a participatory SD approach because it enabled us to compare system behaviour and LPs for change suggested by the archetypes against those identified through the original method (see Box 1). This study draws on the pluralist epistemological perspective described in LIKE, which combined postpositivist and interpretative epistemology (Waterlander *et al*. 2020). In this study, we draw on postpositivism by using empirical evidence to substantiate behaviour over time (also see Step 5). However, due to the complex nature and multistakeholder perspectives in the LIKE data collection (see Box 1), we recognize the value of considering diverse viewpoints, which is why an interpretative epistemology is also necessary.

### Data collection

The data consisted of the LIKE CLD, which was developed through a participatory SD approach (Luna Pinzon *et al*. 2024) (Box 1). We based our analysis on the eight mechanisms identified from this CLD, as these mechanisms included the identified components driving childhood obesity in Amsterdam, allowing us to concentrate on the elements that were previously selected as most important rather than the full CLD. For a full description of these mechanisms, we refer to Luna Pinzon *et al*. (2024). Ethical approval for the data collection of the LIKE project was obtained from the institutional medical ethics committee of Amsterdam UMC, Location VUMC (2018.234). No additional data was collected for this study.

### Data analysis

#### Step one: developing a protocol

We developed a protocol for archetype identification to apply to the LIKE mechanisms based on the seminal SD systems archetype literature (Goodman and Kleiner 1993, Kemeny 1994, Kim and Anderson 1998, Kim 2000a, 2000b, 2000c, Senge 2006, Meadows 2008, Stroh 2015) and guiding principles for qualitative research (Gray 2014). Because there is some discrepancy in the number (and name) of archetypes in the seminal works cited above, we opted first to make a complete list and then compile this into eleven clearly distinguishable archetypes e.g. fixes that fail and success to the successful. The full list including the authors that identified them can be found in Supplementary File 1. For each archetype, we compiled an ‘archetype profile’. The profile encompassed what Kim described as the three properties of an archetype: its (1) storyline (or causal theory), (2) systemic structure (depicted as a CLD with standard configurations of balancing and reinforcing feedback loops), and (3) pattern of behaviour over time [depicted as a graph over time (GoT)] (Kim and Anderson 1998, Kim 2000a, 2000b, 2000c).

#### Step two: identifying systems archetypes in LIKE mechanisms and writing the storylines

Two researchers (J.O. and W.W.) independently searched for the presence of systems archetypes in the original eight LIKE mechanisms (see Box 1). In an iterative way, we interpreted the storyline of the original mechanism, compared it to the archetype profiles and returned to the mechanism, while specifically looking for the underlying ‘deeper’ dynamics for each mechanism and trying to match them with the archetype dynamics. To cross-check whether a mechanism indeed fit an archetype, we used the ‘archetype tree’ developed by Goodman and Kleiner (1993). The archetype tree proposes specific archetype decision paths, e.g. is this mechanism concerned with growth (yes, more unhealthy food); -> does this growth lead to the decline of something else (yes, less healthy food) -> ‘success to the successful’. The two researchers compared their independently identified results, and disputes were discussed until a consensus about the applicable archetype(s) for each mechanism was reached and the archetype storyline was drafted. We were mindful of the possibility that not every mechanism could be reflected in an archetype, but we found that, in this case, we were able to match each of the mechanisms to an archetype.

#### Step three: drawing the archetypes and behaviour over time graphs

We proceeded by completing the archetype profile for each LIKE mechanism with the other two properties: CLD and GoT. We refer to this completed profile as the newly formulated LIKE archetype (LA). The CLDs were drawn by one researcher (J.O.) in Vensim and checked by a second researcher (L.C.). Disputes were discussed until a consensus was reached. In some cases, the storyline (Step 2) was further refined, e.g. by more clearly specifying the ‘fix that failed’ and adding this wording to the storyline itself (i.e. fix, problem symptom, side effect, fundamental solution). Finally, to complete the archetype profiles and to allow cross-checking with empirical data (Step 5), we drew the GoT, identifying the factors that changed over time.

#### Step four: change strategies (leverage points), actions and Intervention Level Framework level

To move from trying to understand system behaviour to identifying fitting LPs, we scouted the archetype literature to identify change strategies that were prescribed for each archetype (Supplementary File 2) (Kim and Anderson 1998, Kim 2000a, 2000b, 2000c, Wolstenholme 2004, Meadows 2008). The archetype literature describes change strategies as blueprints for leveraging change when a certain archetype is at play (e.g. in ‘fixes that fail’: acknowledge that the fix is symptomatic/short term and apply a simultaneous long-term approach), which needs to be ‘translated’ to each specific context. For all newly formulated LIKE archetype (Steps 2 and 3), we formulated context-specific LPs and potential actions, matching the remedy for each archetype as suggested in the literature and the context of the LIKE project. For example, the remedy in the literature for a fix that fails is acknowledging that the fix is failing and implementing actions that target the root cause of the problem. Applying this in the local context meant acknowledging that obesity care (fix) is failing to meet the needs of youth and families, and implementing actions that address the true needs for obesity care while ensuring that changes are sustainably enforced. The LPs and actions were identified by the main researcher (J.O.), refined by two additional researchers (W.W. and L.C.) and finally agreed upon by the entire research team. We placed each LP on the ILF pyramid (from deep to shallow level of leverage): (1) paradigm, (2) goals, (3) system structure, (4) feedback and delay, and (5) structural elements (Johnston *et al*. 2014). This made it possible to compare the newly formulated LPs and actions (and the ILF level) with the original LPs and actions formulated in the LIKE (and the ILF level).

#### Step five: methodological reflections on the structure and traceability of the analysis

We reflected on the process of using systems archetypes as a method, specifically, the reproducibility of the steps compared with the analytical steps undertaken in the LIKE project (Box 1). In addition, to explore how systems archetypes could facilitate cross-checking the causal theory captured in the CLD with existing empirical data and reasoning about the potential effectiveness of a proposed LP, we selected two examples of GoTs for which we sought to identify data points (quantitative for LA5 and qualitative for LA8) that could corroborate whether the current system showed the behaviour over time dictated by the archetype. As the aim in this study was to test how to cross-check behaviour over time using data, we purposefully selected two LAs based on their diverse nature: one that could be cross-checked with national-level quantitative data and one that could be cross-checked with community-level qualitative data obtained as part of the LIKE project. We determined which factors should be supported by data points guided by the GoTs we had drawn for each LIKE archetype (e.g. resources allocated to unhealthy food retailers between 2000 and 2025). The representativeness of both the quantitative and qualitative data was double-checked with experts. The results are presented in the results section Traceable analysis.

## Results

### LIKE archetype profiles: storyline, causal loop diagram, and graph over time

Within the LIKE data, we identified six different systems archetypes (Table 1). ‘Fixes that fail’ was the most common and was observed four times. In three of these four cases, this archetype was combined with ‘shifting the burden’ (twice) or ‘accidental adversaries’ (once). The other identified archetypes were ‘success to the successful’ (observed twice), ‘escalation’, and ‘limits to success’, each observed once.

#### Fixes that fail—resulting in shifting the burden and accidental adversaries:

The ‘fixes that fail’ archetype was observed in LA1, ‘the match between obesity healthcare services and the needs of adolescents with obesity and their parents’, in which the fix of providing care to children (and their families) initially relieves the problem symptom, the need for care. However, this care does not meet the needs of the multiethnic target group in Amsterdam, creating the unintended consequence that families have negative experiences related to obesity care, causing them to avoid it.

In the case of LA2, ‘the use of public outdoor spaces for physical activity, and LA3, livelihood security and poverty’, we observe an additional side effect that directs attention or resources away from the fundamental solution, leading to a ‘shifting the burden’. For LA2, the fix was building outdoor physical activity spaces at the outskirts of town, which were not used, leading to the side effect that there was an impression that physical activity spaces were not needed. This detracts from the fundamental solution of creating (attractive) physical activity space within urban environments. In the case of LA3, the side effect of individual-level health interventions is that health is framed as an individual problem, further detracting from the fundamental solutions addressing poverty and stress.

In the case of LA4 ‘match between local health promotion activities and parents’ needs’, the unintended consequence of the use of health promotion programmes was the development of distrust between parents and health promoters, which is indicative of an ‘accidental adversaries’ archetype.

#### Success to the successful

The ‘success to the successful’ archetype occurs when parties are competing for resources. It was identified in LA5 ‘power dynamics in the current food system and LA6, social norms influencing health behaviours in adolescents’. In LA5, more resources obtained through the high demand and low cost of unhealthy foods led to the success of unhealthy food retailers. This came at the expense of retailers who would like to sell healthy food, which has lower profit margins. Similarly, in LA6, social media attention and marketing resources led to the success of unhealthy social norms. This comes at the expense of attention and marketing resources for healthy content, resulting in its continued decline.

#### Escalation

The ‘escalation’ archetype occurs when one party perceives a threat and acts, followed by a second party reacting, leading to a series of reaction and counter-reaction between the parties that escalates the situation, with both parties being damaged in the process. It was observed in LA7, ‘the role of parents during adolescence’, in which two parties, the parents and adolescents, each took action to counter the perceived threat from the other around the regulation of screen use. The parents sought to regulate screen use, while the adolescents perceived this regulation as a threat and reacted, which in turn led to a counter-reaction from the parents. These actions escalated over time.

#### Limits to success

The ‘limits to success’ archetype occurs when initial growth is stalled or stopped by a ‘constraint’. It was observed in LA8 ‘Connection between health ambassadors (volunteers), municipality and community organizations’, in which, after the initial growth of the health ambassador programme, the lack of support/compensation from the municipality and community organizations limited the further growth of the programme.

### Leverage points, actions, and Intervention Level Framework

When applying the prescribed change strategies to the LIKE archetypes, we observed several differences from the LPs previously identified in LIKE (Table 2), including (1) the need for additional actions; (2) re-shifting action to the root causes; and (3) engaging in different conversations. When placed on the ILF pyramid, five of the newly formed LPs were at the paradigm level, compared to one previously, three at the system goals level, compared to also three previously, and none at the lower system levels, compared to three at the feedback and delay level and one at the structural elements level in the original analysis (Table 3).

**Table 2:** Results of the analysis using the prescribed systems archetypes change strategies to identify leverage points (LPs) and actions.

LIKE archetype (LA)	General change strategy per archetype (theory from the system dynamics literature [see Supplementary File 1] (Kim 2000a, 2000b, 2000c, Wolstenholme 2004, Meadows 2008)	LPs for change, actions, and ILF level as identified in LIKE (Luna Pinzon *et al*. 2024)	Potential LPs for change, actions, and ILF level based on the archetype
**Match between obesity healthcare services and the needs of adolescents with obesity and their parents (LA1)**	**Archetype: fixes that fail** **general change strategies:** Acknowledge the fix is symptomatic/short term, apply a two-pronged strategy to apply the fix while addressing the root problem. In policy resistance, let go of inefficient policies.	**LP**: Increase the experienced support and effectiveness of the obesity care received by families from healthcare professionals. **Action**: Organization-wide scan to identify to what extent the youth healthcare system is sensitive to ethnic diversity. **Action**: Examination room observations. **Action**: Popular scientific article about motivation. **ILF Level 4—feedback and delay**	**Acknowledgement of the fix (symptomatic solution).** It is understandable that health care providers jump to providing care to try to alleviate the growing need for care amongst this group. Acknowledging that the care is not matching the needs of the multiethnic population—and understanding what the needs are—might help to alleviate the unintended consequence (i.e. families have a negative experience and do not want the provided care) and provide care that *does* match the needs. **Two-pronged approach.** In this case, it would be appropriate to continue providing care (the fix) while working to understand how to make the care better match the needs of the families, to avoid the unintended consequence of families with negative experiences related to obesity care. An additional action is to continue to monitor this care, to be sure it *does* meet the needs and expectations, and to avoid a further fix that fails. This acts on the goals of the system by changing the aim from ‘provide care’ to ‘ensure care provided matches the needs and expectations of the patients’ **ILF Level 2—goals**
**The use of public outdoor spaces for physical activity by adolescents (LA2)**	**Archetype: Fixes that fail—Shifting the burden** **General change strategies**: In addition to the fixes that fail change strategies, shifting the burden strategies are: identify the root of the problem, create a shared vision and potentially conduct a ‘painful restructuring’ to break the reinforcing loop that undermines solving the root problem.	**LP:** Adolescents participate in decision-making about the design and organization of public outdoor spaces. **Action:** Co-creation of the public outdoor space. **ILF Level 2—goals**	**Acknowledgement of the fix (symptomatic solution)**. The fix (e.g. building physical activity spaces on the outskirts) is being applied to attempt to increase physical activity amongst adolescents. Acknowledging that the fix is a symptomatic solution helps to identify the root (e.g. there is a lack of appropriate physical activity space in urban areas/space in the city remains scarce). This may be related to a fundamental choice to prioritize housing and business over outdoor spaces for physical activity. Action at a deeper level of the system to address this fundamental problem still lacks. Change strategies from ‘shifting the burden’ also include creating a shared vision (e.g. how should urban spaces facilitate physical activity). Bringing stakeholders together (e.g. young people, policy makers, developers) to discuss and find a shared vision around physical activity (space) in urban areas might help stakeholders to look for solutions of providing attractive outdoor public spaces within the urban setting. **Let go**. Hammering away at the fix (building physical activity spaces on the outskirts) and trying whatever possible to make them work is bound to fail. Taking a step back, a deep breath and re-looking at the vision might help the stakeholders to better formulate ideas and actions to readjust how we create spaces in urban areas. Shifting to the root cause would include re-envisioning urban design to support daily physical activity, recognizing that simply providing designated spaces without integrating movement throughout the day is insufficient—this acts on the system’s belief. **ILF Level 1—paradigm**
**Livelihood security and poverty (LA3)**	**Archetype: fixes that fail—shifting the burden** **general change strategies**: In addition to the fixes that fail change strategies, shifting the burden strategies are: identify the root of the problem, create a shared vision and potentially conduct a ‘painful restructuring’ to break the reinforcing loop that undermines solving the root problem.	**LP:** Health is included as an important topic in policies that relate to social security. **Action**: Connecting health and livelihood security. **ILF Level 2—goals**	**Acknowledge the fix that fails** e.g. that health programmes are ineffective at reversing the health trends in people with low socioeconomic status. This acknowledgement might be difficult/painful, as time, energy and resources have been poured into these health programmes. Meadows refer to this as a painful restructuring. It is not easy to get out of this loop (and better to avoid getting into it). However, there is a need to completely refocus resources away from programmes that frame the responsibility of health as that of the individual and towards work that the root causes of health problems such as poverty and stress. This needs to be made explicit to the public as well as to health workers, who will otherwise continue with symptomatic and short-term solutions. A two-pronged approach may not be effective in this scenario because the ‘fix’ is also resulting in shifting the burden reinforcing loop. It drives the frame that health is an individual responsibility. Rather, it calls for the painful restructuring approach as described above. This acts at a paradigm level by working to address root causes of poverty and stress and challenging core beliefs around whom is responsible for health (individual or society). **ILF Level 1—paradigm**
**Match between local health promotion activities and parents’ needs (LA4)**	**Archetype: Fixes that fail—accidental adversaries** **general change strategies:** acknowledge the fix that failed and recognize that the damage is done unintentionally. Accidental adversaries recognize there is a lack of coordinated action between two or more parties.	**LP**: Local health promotion activities meet parents’ needs. **Action idea**: Case study health promotion activity called ‘parenting debates’. **ILF Level 4—feedback and delay**	Overcoming an ‘accidental adversaries’ archetype requires strengthening an understanding of the partner’s fundamental needs, and recognition that damage is done unintentionally. We might envision an action that helps to **repair the trust** between the parents and the local health professionals. If it is not possible to repair the trust, it might be necessary to recruit other (trusted) individuals to fill this role. For example, local volunteers, municipal workers or researchers. This acts on the goals of the system that are now focused on providing health promotors, to ensuring that there is trust between those providing health information and those receiving it. **ILF Level 2—goals**
**Power dynamics in the current food system (LA5)**	**Archetype: Success to the successful** **General change strategies**: level the playing field (break up concentration of power), make competitors collaborators and support the ‘weaker’ side of the equation.	**LP**: Supermarkets and schools take joint responsibility for the role they play in shaping adolescents’ food environment. **Action**: Group model building workshops with food retailers and/or schools. **LP**: Policies that increase and support the availability and accessibility of healthy food. **Action**: Exposing retails tactics and lack of action in obesity prevention. **Action**: Developing an active lobbying initiative between academia and policy practice **Action**: Entrepreneur network **ILF Level—2 goals**	**Level the playing field** for example by correcting the balance between the availability of healthy vs. unhealthy food (e.g. in the availability of healthy vs. unhealthy foods). For example, the municipality could set a policy with a mix of healthy and unhealthy (e.g. shops/stores within a shopping street or neighbourhood). Through doing so the municipality regulates the reinforcing loop and breaks the mechanism that the availability of unhealthy dominates. **The concentration of power** is currently in the hands of the unhealthy food retailers. In order to break this up, it might be necessary for local or national government to intervene. One path to reach intervention is lobbying to expose retail tactics or practices that concentrate the power in one group. Antitrust measures could help to break up the concentration of power in the food retail sector. Further, **levelling the playing fiel**d through strategies such as taxes on unhealthy foods, or true pricing strategies could help to reduce the resources available for the unhealthy food retailers. Helping the healthy food entrepreneurs is another strategy, but only if their business model does not reflect profit maximization (then you revert to another success to the successful model). This acts at the paradigm level on the core beliefs in a profit-driven economic system. **ILF level 1—paradigm**
**Social norms influencing health behaviours in adolescents (LA6)**	**Archetype: success to the successful** **general change strategies**: level the playing field (break up concentration of power), make competitors collaborators and support the ‘weaker’ side of the equation.	**LP**: A new social norm in which exhibiting healthy behaviours is considered cool and normal. **Action**: Peer role models **Action**: Role models network of youth workers and adolescents. **ILF Level 1—paradigm**	**Level the playing field** by breaking up the concentration of power by unhealthy. This could be some type of regulation (e.g. banning unhealthy food content on social media for adolescents) to give more space for ‘healthy’ to receive more attention. Promote or support influencers to promote healthy norms on social media—but also to attack unhealthy. Examples are influencers who expose the lies in the videos of other (health) influencers, taking it apart piece by piece and backing it up with science. This challenges core beliefs and norms around healthy behaviour, acting at a paradigm level. **ILF Level 1—paradigm**
**The role of parents during adolescence (LA7)**	**Archetype: escalation** **general change strategies:** avoid the situation before it starts and address deep-rooted assumptions in the perception of threat and control.	**LP:** Parents can set, monitor, and enforce rules regarding sleep, dietary, screen, and physical activity behaviour. **Action**: Rules Rule—workshop in which parents who serve as health ambassadors in the neighbourhood learn about parenting skills from a professional and from each other and how to set and enforce rules. The insights gathered during the workshop will be transferred to short vlogs, which will be disseminated to other parent. **ILF Level 4—feedback and delay**	**Avoid** a situation of escalation by addressing the threat and control at the early ‘tit-for-tat’ stages. This could involve changing the discussion around control of screen time at the ‘early stages’ when an adolescent begins using a screen and, for example, acknowledging that parents also lack self-control on their screens and potentially finding a common path such as putting the phones in a box together. **Addressing the deep-rooted assumptions—**in this model of escalation, we assume that it is the parental job to control phone use but also that the adolescent will feel threatened and react. Acknowledging this might help to have a different conversation around setting rules within a household (but also broader in society). This model emphasizes the parent–adolescent relationship, but we can also look at this from a society-wide perspective. What do we need as a society to combat this type of escalation (e.g. the parental pact on age for telephones helps take the responsibility away from the parent from the perspective of the teenager—which may lower the perceived threat of the parent to the adolescent). This acts at a paradigm level on the core beliefs about where responsibility lies in terms of screen regulation for teenagers. **ILF Level 1—paradigm**
**Connection between health ambassadors (volunteers), municipality and community organizations (LA8)**	**Archetype: limits to success** **general change strategies:** anticipate issues to scaling ahead of time and relieve pressures in the system.	**LP:** Improve the commitment of health ambassadors to influence local residents towards a healthy lifestyle. **Action**: Interviews with health ambassadors about their role. **ILF Level 5—structural elements**	Limits to success are often seen in the scaling of community-based health initiatives. In this archetype is best to **anticipate issues related to scaling** ahead of time. However, in this example, that was not done. The next strategy is to **relieve pressures on the system** by, for example, the limiting action or the constraint. Actions should focus on high workload of the ambassadors and the constraint from the municipality and community organizations. The municipality could be consulted to understand why the constraint exists and what type/how much support they can give. Additionally, health ambassadors could be relieved of some of their responsibilities (duties done by a third party or the performance of the ambassadors declines). Relieving the pressure on the system by removing the limiting constraint acts on the goals of the system. **ILF Level 2—goals**

**Table 3:** The Intervention Level Framework (ILF) levels, definitions of each level and the number of leverage points (LPs) identified in the original analysis as compared with the archetype analysis.

ILF level	Definition (Luna Pinzon *et al*. 2022, Stronks *et al*. 2024)	Original LIKE LPs	Newly formulated LPs
Paradigm	The system’s deepest held belief	1 (LA6)	5 (LA2, LA3, LA5, LA6, LA7)
Goals	The final outcomes that the system strives towards	3 (LA2, LA3, LA5)	3 (LA1, LA4, LA8)
Structure	Subsystems derived from feedback loops and interconnections between components	0	0
Feedback and delay	Self-regulation of the system to support the deepest held belief	3 (LA1, LA4, LA7)	0
Structural elements	Actors, subsystems and physical elements	1 (LA8)	0

#### The need for additional actions

The need for additional actions was observed in four cases: LA1, ‘the match between obesity healthcare services and the needs of adolescents with obesity and their parents; LA4, match between local health promotion activities and parents’ needs; LA5, power dynamics in the current food system; and LA6, social norms influencing health behaviours in adolescents’. In LA1, the ‘fixes that fail’ change strategy acknowledges that the fix fails due to mismatched needs and applies a two-pronged approach that continues to provide care while resolving mismatched needs. In the LIKE project, the actions focused on understanding the needs and expectations of the families and introducing an assessment of care provider’s diversity-responsiveness (both ILF level 4/5—feedback and delay). The archetype analysis prescribes an additional action, which is to continually assess if progress or changes have been made so that the care *does* meet the needs and expectations of the families, to avoid a further fix that fails. This additional action acts on the goals of the system (ILF level 2/5—goals).

The ‘accidental adversaries’ archetype in the case of LA4 highlights the underlying problem of unintended betrayal, which involves trust. The action developed in LIKE aims to better understand parents’ needs and wishes (ILF level 4/5—feedback and delay), which is part of the change strategy of this archetype. However, asking additional time from parents without being able to make changes could exacerbate the fix that fails. Additional actions would focus on rebuilding trust between health workers and parents or recruiting trusted individuals to fill this role, which would target the goals of the system itself (ILF level 2/5—goals).

The need for additional action strategies was also observed in both of the ‘success to the successful’ LIKE archetypes (LA5 & LA6). In both cases, the actions developed during LIKE focused on supporting the ‘weaker’ side (e.g. policies to increase the availability and accessibility of healthy food (LA5) (ILF level 2/5—goals) and support for social norms influencing healthy behaviour (LA6) (ILF level 1/5—paradigm)). However, the archetype indicates that actions are also necessary to break up the concentration of power and resources that favour the ‘successful’ half of the archetype to level the playing field. In the case of LA5, this would include breaking up the concentration of power by exposing retail tactics or antitrust measures in the food sector, challenging the existing paradigm around profit maximization (ILF level 1/5—paradigm). In the case of LA6, regulating or breaking up the power concentration of unhealthy influence (e.g. banning unhealthy food content on social media) while increasing the influence of healthy content (ILF level 1/5—paradigm).

#### The need to reshift focus to root causes

The need to reshift focus to root causes was observed in three LIKE archetypes: LA2, ‘the use of public outdoor spaces for physical activity; LA3, livelihood security and poverty; and LA8, the connection between health ambassadors (volunteers), municipality and community organizations’. In LA2, the action developed in LIKE was to co-create activity spaces with adolescents (ILF level 2/5—goals). However, this is a symptomatic solution because even if the co-created spaces would be more attractive, they would still be on the outskirts of town, which was the critical factor for lack of use. Shifting to the root cause would include re-envisioning urban design to support daily physical activity, recognizing that simply providing designated spaces without integrating movement throughout the day is insufficient, which would act on the system’s belief (ILF level 1/5—paradigm).

Similarly, in LA3, the action ideas aimed to connect policy areas of health and social services, which acted on changing the goals of the system (ILF level 2/5—goals). If archetype change strategies are followed, drastic steps would be necessary to break the reinforcing loop of health framed as an individual problem, including ‘painful’ restructuring to target the root causes and change the system’s belief (ILF level 1/5—paradigm). Actions could refocus resources away from individual health programmes and those who would otherwise continue with symptomatic, short-term solutions.

Finally, in LA8, the root cause of the constraint was not addressed. The action idea in LIKE to interview health ambassadors regarding their needs and wishes (ILF level 5/5—structural elements) does not fit with the prescribed changed strategy. Rather, the archetype change strategy points to target the ‘constraint’. This means understanding what compensation and support the municipality and community organizations could provide to relieve pressure in the system, thereby targeting the system’s goals (ILF level 2/5—goals).

#### The need to engage in different conversations

The need to engage in different conversations was observed in LA7: ‘the role of parents during adolescence’. The educational modules developed in LIKE aimed at improving parental skills regarding screen use (ILF level 4/5—feedback and delay). This approach does not challenge the belief that parents are ultimately responsible for children's screen use and associated harm. The escalation archetype shows that we could engage in a different type of conversation to avoid escalation in the first place and address deep-rooted assumptions. For example, is it the parent's responsibility to determine the telephone age for children, or how much screen use is appropriate? Broader societal agreements could help remove responsibility from parents and de-escalate the parental ‘threat’ in the situation, targeting paradigms (ILF level 1/5—paradigm).

### Using systems archetypes for a structured and traceable analysis

#### Structured analysis

Tables 1 and 2 show how we crafted templates that allowed for the structured analysis of the mechanisms, starting with the storylines, then the CLDs, the GoTs and finally the change strategy. Each step used the guiderail provided by the archetype. The CLDs and GoTs, as given by Kim and others, prescribe factors that we identified in each storyline (i.e. problem symptom, fix or resources). Each mechanism could be systematically broken down into these factors over time. At each step, we were forced to critically reflect if the structure matched the behaviour over time and if we truly reached the most fundamental causes. This approach was more structured than our original participatory approach, which used collaborative group discussions, to reason about how factors, feedback loops and subsystems may contribute to system behaviour without clear anchors to guide the discussion. The discussions lacked the language provided by the systems archetypes, which made it difficult to bring the discussion from separate subsystems to the higher level of overall system behaviour and to make hypotheses about the link between system structure and system behaviour explicit.

We found two ways in which the archetypes refined our analysis: (1) the LIKE mechanism ‘fit’ with an archetype with little to no modification, where the archetype mainly helped to sharpen the storyline (and develop additional/different LPs) (LIKE mechanisms M1, M5, M6, M7, M8); (2) LIKE mechanisms that, as it turned out, were formulated in terms of system structure only and therefore needed significant modification to formulate the storyline in a behaviour-driven way (while still staying close to the original data) (M2, M3, M4). For example, in LA2 the original mechanism (M2 in LIKE) was formulated as:

**The use of public outdoor spaces for physical activity by adolescents**. In highly populated cities such as Amsterdam, the high demand for housing and business has resulted in public outdoor spaces designed for active play and sports being built on the outskirts of neighbourhoods. Larger distances to such spaces are a barrier for adolescents. Furthermore, public outdoor spaces are generally unattractive for adolescents as they are often designed for younger children, or adults, or for one specific activity, for example, basketball or soccer. Furthermore, adolescents are not engaged in the decision-making/design/organization of public outdoor spaces, resulting in these spaces not matching their wishes and/or needs.

It was modified to, from the perspective of fixes that fail:

‘The problem is that adolescents are not physically active (enough), leading to the problem symptom ‘the need to do “something”’ about adolescent inactivity in urban areas. The fix is building physical activity locations at the edge of the city. That fails because it is known that an important aspect of physical activity is daily practice, which comes from active transport, playing outside, etc., rather than traveling to a different neighbourhood for access to physical activity spaces (fix that fails). The side-effect is that the amenities built at the outskirts are not being used, leading to the impression that there is no need/no interest, which leads to even less investment in these kinds of amenities (in places in which they would perhaps be used) and reducing the attention and resources for the fundamental solution of creating appropriate physical activity (close to home) in urban areas (shifting the burden).’

The two versions of the mechanisms are presented in Table 1.

#### Traceable analysis

We collected data to analyse the GoTs for two of the LIKE archetypes (LA5 & LA8). Using the data points for the variables derived from Kim, we were able to check whether the behaviour over time prescribed by the archetypes matched with the actual observed past behaviour over time by cross-referencing with qualitative and quantitative data. In both cases, the data were in line with the behaviour over time prescribed by the archetype. The results are presented in Boxes 2 & 3.

Box 2 Quantitative empirical data example: comparing the graphs over time prescribed by the archetype with actual observed data over time. **Power dynamics in the current food system (LA5)**

Based on Kim’s ‘success to the successful’ GoT, we identified five variables for which we should try to collect data points that show past trends over time revolving around the resources that were allocated to unhealthy versus healthy retailers and the success attained by unhealthy versus healthy retailers. On the ‘resources’ side we looked at (1) consumer food choice (allocated to unhealthy versus healthy food), (2) lower prices of unhealthy food as compared to healthy food (through high demand, leading to more resources for distribution, marketing and development) and (3) marketing budgets (which could be used to indicate how many ‘resources’ each food retailer had to spend). On the ‘success’ side we looked at (4) how many unhealthy versus healthy food retailers there were over time (i.e. the density of retailers). For resources as well as success, we looked to find data corroborating that allocation to one came at the *expense* of the other. Finally, we describe (5) the general food consumption trend specific to the Netherlands, as outlined in a report.

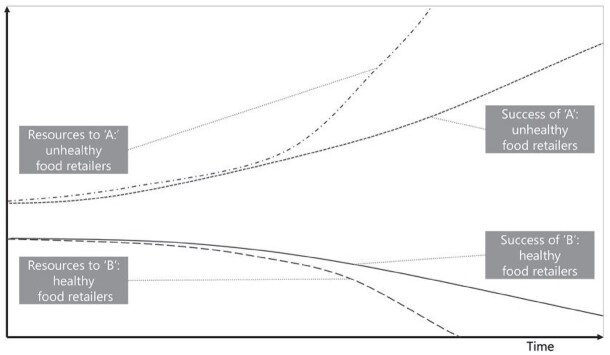

Data over time showing the resources allocated to unhealthy food at the expense of healthy food

- (1) Consumer food choice (allocated to unhealthy versus healthy food): Recent research from the United States shows that consumers choose unhealthy foods at the expense of healthy foods. ‘Ultra-processed foods displace more nutritious foods in diets, such as fruits, vegetables, legumes, nuts, and seeds’ (Lane *et al*. 2024). This has been shown to occur over time, ‘the evidence from analyses of nationally representative data sets collected in 11 countries from 2001 to 2015 shows that the displacement of non-ultra-processed by ultra-processed foods is consistently associated with an overall deterioration of the nutritional quality of diets’ (Monteiro *et al*. 2019).
- (2) Lower prices of unhealthy food as compared to healthy food (through high demand, leading to more resources for distribution, marketing and development): In the Netherlands, the price of unhealthy food increased *less* than the price of healthy food, 15% compared to 21%, respectively from 2010 to 2020 (CBS 2021).
- (3) Marketing budgets (which could be used to indicate how many ‘resources’ each food retailer had to spend): Marketing for unhealthy food was 4.2 billion from 2018–2024, compared to 640 million for healthy food in the Netherlands (de Beus and ter Wijden 2024).

Data over time showing the success attained by unhealthy food retailers at the expense of healthy food retailers from 2004 to 2017 in the Netherlands (Pinho *et al*. 2020)

(4) How many unhealthy versus healthy food retailers there were over time (i.e. the density of retailers):

- Density of food retailers per 10 000 inhabitants remained relatively constant from 2004 (35.7) to 2017 (35.8)
- Density of fast food restaurants (unhealthy food retailers) increased by 14.6%
- Density of local food shops* (healthy food retailers) decreased by 26.3%

(5) The general food consumption trend specific to the Netherlands: A report from the Netherlands National Institute for Public Health and the Environment describes the general trend from the 1990s to 2016 that Dutch consumers purchase more food in supermarkets as compared to local food retailers (e.g. butchers, bakers, greengrocers). They consume more convenience and processed foods. The supply chain favours big retailers such as supermarkets (Geurts *et al*. 2017).

*local food shops as a proxy for ‘healthy’ retailers as their main provisions included: potatoes, vegetables, fruit, meat and meat products, poultry, bread and pastries, fish, crustaceans and molluscs.

Box 3 **Qualitative empirical data example: comparing the graphs over time prescribed by the archetype with actual observed data over time**. **Connection between health ambassadors (volunteers), municipality and community organizations (LA8)**

Based on Kim’s ‘limits to success’ GoT, we identified three variables for which we should try to collect data points, that show the past situation and past trends over time: (1) the effort to increase the number of active volunteer ambassadors, (2) the ‘constraint’ of the municipality or organizations and (3) the performance (or ability/capacity) of the volunteer ambassadors to perform their role. We were able to look at data from two sources. First, we were able to find a related quote describing behaviour over time in the qualitative data that were initially collected in LIKE. Second, we were able to directly ask questions related to the behaviour over time to the field researcher that had been responsible for collecting these qualitative data in LIKE.

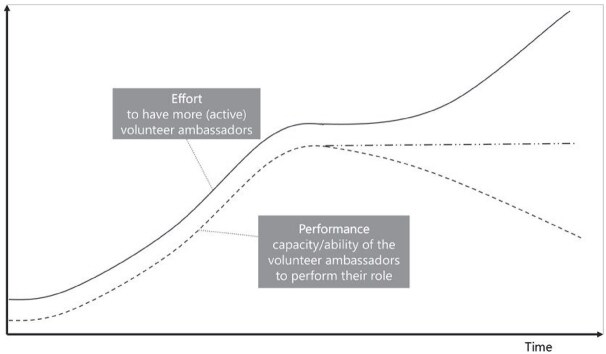

**Quote from LIKE data:**

‘The approach currently being followed is successful in the sense that a large number of volunteers are available and trained (approximately 200 in the past year). The health ambassadors also organize successful activities that residents want to participate in. However, at the same time, we must acknowledge that the number of individuals who continue to actively commit themselves as health ambassadors is limited. As a result, the active health ambassadors feel that the work that needs to be done rests on the shoulders of a (too) small group’.

**Questions asked to field researcher:**

1. Did the performance of the volunteer ambassadors increase over time?
  I did not track this. I think it also depends on whose perspective you consider. No clear answer.
2. Was there any reason the performance of volunteer ambassadors would be constrained such as lack of support from the municipality or organizations?
  Yes.
3. Did this lead to a reduced number of volunteer ambassadors, less capacity or a decrease in activities?
  It led to fewer ambassadors and reduced capacity. A decrease in activity depends on the perspective again. Activities carried out in the name of the municipality may have decreased.
4. Is there reason to believe that the municipality expected that training more volunteer ambassadors would automatically lead to an increase in the number of active volunteer ambassadors?
  Yes.
5. And that this approach initially worked (more trained volunteer ambassadors = more active volunteer ambassadors)?
  Yes.
6. However, later the municipality encountered an unexpected limitation or barrier?
  Yes, fewer ambassadors committed themselves (even though they had been trained).

## Discussion

This study aimed to explore if, in comparison with the previous analysis of the LIKE project, the use of system archetypes (1) helped to understand the behaviour of the system, (2) allowed for the development of LPs and actions that could drive change at deeper levels of the system (according to the ILF), and (3) provided a more structured and traceable analysis that linked system structure to system behaviour and ultimately LPs for change. In our *post hoc* analysis of previously identified mechanisms in the participatory SD LIKE project, we observed six different systems archetypes. The archetype ‘fixes that fail’ was the most common, followed by ‘success to the successful’, ‘escalation’, and ‘limits to success’. Compared with the original mechanisms, which pointed to specific relationships (e.g. the unfair competitive position between healthy and unhealthy foods), the archetypes provided a more overarching storyline by capturing recurring SD (e.g. framing this as a case of ‘success to the successful’). From here, we could systematically identify LPs that specifically target system behaviour. The new LPs required introducing new action strategies, reformulating approaches to address root causes, and engaging in different conversations. This also positioned the LPs deeper within the system, meaning they have a greater ability to leverage system change, according to the ILF levels. Finally, upon reflecting on the use of systems archetypes as a methodology, we show that it structured the process, which limited researcher interpretation and improved reproducibility. Empirical data was also applied to the GoTs to cross-check the hypothesis that a particular archetype is at play in the system by comparing the archetype’s GoT to historical data describing the system's past behaviour. This has implications for evaluation, with the potential to observe changes in the trend lines following the implementation of a corrective action.

Systems archetypes provide a structured method for compiling a full profile of the archetype in a specific context, including storyline, CLD and GoT, which ‘forces’ researchers to follow guiderails such as naming the ‘fix’ that failed or ‘unintended consequences’ of interventions. This differs from the traditional participatory methods which tend to rely on group discussions to hypothesize these mechanisms. In our analysis this helped us to understand the system behaviour by better describing the underlying dynamics (e.g. the healthcare ‘fix’ fails when it does not meet the needs of the multiethnic target group and causes unintended consequences of negative experiences) rather than the events we observe happening (e.g. healthcare services do not meet the needs of the multiethnic target group). As Kim explains, the use of systems archetypes helps one observe recurring dynamics rather than focusing on individuals or events (Kim and Anderson 1998). Subsequently, by providing blueprints for change strategies, systems archetypes again give guiderails for pinpointing LPs for change that are by definition deeper in the system. This guided approach also brought *common* system traps to the surface, highlighting, in this case, the predominance of the ‘fixes that fail’ archetype.

The predominance of the ‘fixes that fail’ archetype is somewhat logical in public health. Public health promoters, health insurance companies and stakeholders are looking for solutions to address public health problems, such as build physical activity spaces or provide care to adolescents with obesity. However, they are often constrained by the need for tangible results as required by the existing structures (e.g. 5-year research projects, government election cycles, or health insurance budgets) which can result in policy inertia (Hagenaars *et al*. 2024). According to the ‘fix that fails’ archetype, investing in interventions that only scratch the surface of a problem might inherently undermine the necessary long-term interventions when efforts to temporarily ‘fix’ a problem's symptom, take our eyes off underlying causes (‘shifting the burden’), when they reduce the expectations (‘drifting goals’), or create situations of distrust (‘accidental adversaries’). Applying this to the LIKE project, the systems archetypes analysis of the ‘fix that fails’ scenarios revealed that three of the four original LPs risked ‘perpetuating’ the ‘fix that fails’ by addressing symptoms instead of root causes. However, since we had a deeper understanding of system behaviour and predefined change strategies, we used these as blueprints to identify deeper LPs and find a way out of the ‘fix that fails’ cycle. While systems archetypes cannot solve short-term projects and results-driven policy making, they might help to provide a language to describe the phenomenon and more quickly arrive at actionable ‘deeper’ change strategies. They could help avoid (or at least be cognizant of) unintended consequences in the future, such as landing in another fix that fails. We propose that systems archetypes can be applied to future research and in (policy) practice to help understand system behaviour and identify LPs for change.

This study contributes to the limited research applying systems archetypes in public health. Although interest is growing, in our forthcoming review on systems archetypes we found that few studies have systematically used them as an analytical lens from inception to conclusion. Two recent notable studies use systems archetypes as an ‘anticipatory’ framework to analyse potential unintended consequences of interventions (Alvarado *et al*. 2023, Milton *et al*. 2024). Milton *et al*. (2024) applied them to intervening in the complex system around physical inactivity, whereas Alvarado *et al*. (2023) used them to reflect on patterns in a CLD, describing the intervention of street trees and its potential effects on mental health. Both studies used the archetypes to avoid ‘just’ prescribing an intervention: they try to interpret *how* the intervention might interact within the system over time and identify potential (unintended) consequences. Milton *et al*. (2024) concluded that ‘adaptive policies are particularly appropriate when it is necessary to avoid or counter unwanted patterns of behaviour of a dynamic system, as characterized by the archetypes’. Our findings add that systems archetypes help *structure* the analysis by providing complete profiles for each archetype. We also demonstrate how this can be used to cross-check behaviour over time using both qualitative and quantitative data. This process forces the modeller to be explicit about assumptions embedded in their model (e.g. the success to the successful type assumes that resources allocated to unhealthy food come at the expense of resources allocated to healthy food), where they can then examine whether these assumptions are supported by empirical evidence. It facilitates the integration of empirical data in systems approaches to public health and has implications for evaluating these methods.

### Limitations and recommendations

One limitation of our study is that we conducted this analysis purely based on researcher review of the data from the original analysis rather than including perspectives from practitioners, policymakers, or the target group themselves. Our initial goal was to test and develop this methodology further; however, ideally, we would involve other stakeholders in the analysis, as they may offer different perspectives on the applicability of the archetypes and suggested actions. Additionally, this analysis was performed *post hoc*, meaning that we only theorized about the effects these actions could have. This type of analysis could instead be applied in the action-forming phase (e.g. during the group model-building sessions), including an assessment of the feasibility of implementing the actions. Finally, while we tested the achievability of cross-checking the behaviour over time graphs with empirical data, this was limited to two examples. Examining the real-world behaviour over time of the additional six LAs was beyond the aim of this paper. We suggest that future research tests archetype-informed LPs in real-world interventions. However, we are not arguing that systems archetypes are necessarily a suitable method for all systems approaches in public health. An important methodological consideration, as we identified in our forthcoming review on systems archetypes, is that systems archetypes emphasize specific aspects of system behaviour: they help look for a specific set of patterns of behaviour, but there can be other important characteristics of system behaviour that fall outside this scope (Kim and Anderson 1998, Senge 2006, Meadows 2008). As such, not all complex public health problems studied using SD methods may reflect a systems archetype (Davis *et al*. 2018). Therefore, the use of systems archetypes should be approached with care and consideration.

## Conclusions

Our findings show that systems archetypes could be a much-needed stepping stone to move beyond merely showing a system’s structure in (participatory) SD approaches to more structured reasoning about the behaviour that the system structure in question is producing and how to change it. Our findings also show that archetypes help identify LPs for change and actions at the deepest levels of the system. Based on these experiences, we contend that systems archetypes can be a valuable tool in providing a more structured and traceable analysis of system behaviour, helping researchers and practitioners analyse and change complex systems in public health. Future research should test whether the application of systems archetypes leads to more effective actions in practice.

## Supplementary Material

daag056_Supplementary_Data

## Data Availability

The datasets supporting the conclusions of this article are included within the article and its supplementary files.

## Abbreviations

CLD: Causal Loop Diagram
GMB: Group Model Building
GoT: Graph over Time
ILF: Intervention Level Framework
SD: System dynamics
LP: Leverage point
