# Supplementary material for: Using systems archetypes to understand system behaviour and identify leverage points for change in local obesity prevention in The Netherlands
Source: Health Promot Int. 2026 May 19;41(3):daag056. doi: 10.1093/heapro/daag056 (PMC13187847; doi:10.1093/heapro/daag056)
Supplement: daag056_Supplementary_Data [file daag056_supplementary_data.zip › Supplementary File 1.pdf]

Supplementary File 1

List of systems archetypes as found in the seminal system dynamics literature

Number	Archetype (generic name)	Identified in/by:<br><br>Kim (1-4), Meadows (5) , Stroh (6), Goodman & Kleiner (7), Kemeny (8)
1	Success to the Successful	Kim, Meadows, Stroh, Goodman & Kleiner
2	Fixes that fail	Kim, Meadows, Goodman & Kleiner, Stroh
3	Shifting the burden	Kim, Meadows, Goodman & Kleiner, Stroh
4	Limits to success	Kim, Goodman & Kleiner, Stroh
5	Drifting Goals	Kim, Meadows, Goodman & Kleiner, Stroh
6	Growth and Underinvestment (fixed standards)	Kim, Goodman & Kleiner, Stroh
7	Growth and Underinvestment (drifting standards)	Goodman & Kleiner
8	Escalation	Kim, Meadows, Goodman & Kleiner, Stroh
9	Tragedy of the Commons	Kim, Meadows, Goodman & Kleiner, Stroh
10	Accidental Adversaries	Goodman & Kleiner, Kemeny, Stroh
11	The Attractiveness Principle (Competing Goals)	Goodman & Kleiner, Stroh

References

1. Kim DH. Systems Archetypes I: Diagnosing Systemic Issues and Designing High-Leverage Interventions. 2000.
2. Kim DH. Systems Archetypes II: Using Systems Archetypes to Take Effective Action 2000.
3. Kim DH. Systems Archetypes III: Understanding Patterns of Behavior and Delay. 2000.
4. Kim DH, Anderson V. System Archetype Basics Waltham, Massachusetts: Pegasus Communications, Inc; 1998.
5. Meadows DH. Thinking in systems: A primer: chelsea green publishing; 2008.
6. Stroh DP. System Thinking for Social Change Chelsea Green Publishing Co; 2015.
7. Goodman MR, Kleiner A. Using the Archetype Family Tree as a Diagnostic Tool. Cambridge, MA: Pegasus Communications Inc; 1993.
8. Kemeny J. "Accidental Adversaries": When Friends Become Foes. The Systems Thinker1994.
