# Supplementary material for: Using systems archetypes to understand system behaviour and identify leverage points for change in local obesity prevention in The Netherlands
Source: Health Promot Int. 2026 May 19;41(3):daag056. doi: 10.1093/heapro/daag056 (PMC13187847; doi:10.1093/heapro/daag056)
Supplement: daag056_Supplementary_Data [file daag056_supplementary_data.zip › Supplementary File 2.pdf]

Supplementary File 2

List of change strategies identified from seminal system dynamics literature per identified systems archetype

Archetype	Change strategies
Success to the successful	Kim – Archetype Basics (1)<br>• Look for reasons why the system was set up to create one winner<br>• Chop off one half of the archetype by focusing efforts and resources on one group<br>• Find ways to make teams collaborators rather than competitors<br>• Change systems so that collaboration rather than competition gets rewarded, invest in under-resourced groups, prioritize societal over individual goals (e.g., social cohesion rather than self-actualization).<br><br>Kim – Archetypes I (2)<br>• "What is the larger goal?"<br>• "Lop off" one side by determining ahead what the winner should be and putting all resources there, rather than using competition to drive a winner and a loser.<br>• Make competitors collaborators<br>• An environment in which all can provide equally (without competition).<br><br>Kim – Archetypes II (3)<br>• Look for historical & competency traps (if it's 'always been done that way'). It may be a case of the first not the fittest.<br><br>Kim – Archetypes III (4)<br>• It is about inertia – after you reach a 'tipping' point it will continue going to one party<br>• It's easier to maintain than to change<br>• "We may end up with a person/activity/product not because it's the fittest but because it was the first or the most widely-available"<br>• Understand how the success of A might systematically undermine B – and how to uncouple the two<br>• Look beyond what works 'now' and to what we really want to do.<br><br>Meadows (5)<br>• "Species and companies sometimes escape competitive exclusion by diversifying. A species can learn or evolve to exploit new resources. A company can create a new product or service that does not directly compete with existing ones"<br>• "The success-to-the-successful loop can be kept under control by putting into place feedback loops that keep any competitor from taking over entirely. That's what antitrust laws do in theory and sometimes in practice."<br>• "The most obvious way out of the success-to-the-successful archetype is by periodically "levelling the playing fi eld." Traditional societies and game designers instinctively design into their systems some way of equalizing advantages, so the game stays fair and interesting"

	• "Diversification, which allows those who are losing the competition to get out of that game and start another one; strict limitation on the fraction of the pie any one winner may win (antitrust laws); policies that level the playing fi eld, removing some of the advantage of the strongest players or increasing the advantage of the weakest; policies that devise rewards for success that do not bias the next round of competition"<br><br>Wolstenholme (6)<br>• Adding a 'regulatory' link to break the advantage for A, overtime increasing the relative outcome for B.<br>• See Figure B3.
Fixes that Fail	Kim – Archetype Basics (1)<br>• "Acknowledge that the fix is merely alleviating the symptom and make commitment to solve the real problem"<br>• "Two-pronged attack of applying the fix and planning out fundamental solution to not get caught in the cycle."<br><br>Kim – Archetypes I (2)<br>• Same as Archetype basics<br><br>Kim – Archetypes II (3)<br>• Define the problem symptom (watch out that it's not the problem solution) e.g. "lack of sales training"<br>• Map current interventions or past 'solutions'<br>• Map unintended consequences – potential side-effects<br>• Identify loops that create problem symptoms – it can be hard to recognize the fundamental cause<br>• Identify high-leverage interventions that address the root problem<br>• Map potential side effects (so you can get ahead of the curve)<br><br>Kim – Archetypes III (4)<br>• "If you suspect you might be caught in a fixes that fail dynamic, look for repeating patterns of quick fixes, determine how often these fixes occur, and compare that to the frequency with which you typically review performance"<br><br>Meadows (5)<br>• "The alternative to overpowering policy resistance is so counterintuitive that it's usually unthinkable. Let go. Give up ineffective policies. Let the resources and energy spent on both enforcing and resisting be used for more constructive purposes. You won't get your way with the system, but it won't go as far in a bad direction as you think, because much of the action you were trying to correct was in response to your own action. If you calm down, those who are pulling against you will calm down too. This is what happened in 1933 when Prohibition ended in the United States; the alcohol-driven chaos also largely end."<br>• "The most effective way of dealing with policy resistance is to find a way of aligning the various goals of the subsystems, usually by providing an overarching goal that allows all actors to break out of their bounded rationality. If everyone can work harmoniously toward the same outcome (if all feedback loops are serving the same goal), the results can be amazing."

	• "Let go. Bring in all the actors and use the energy formerly expended on resistance to seek out mutually satisfactory ways for all goals to be realized—or redefinitions of larger and more important goals that everyone can pull toward together."<br><br>Wolstenholme (6)<br>• See Figure B2.
Shifting the Burden	Kim – Archetype Basics (1)<br>• Problem symptoms are usually easier to recognize than the other elements of the structure<br>• If the side-effect has become the problem you may be dealing with an addiction structure<br>• Whether a solution is "symptomatic" or "fundamental" often depends on one's perspective. Explore the problem from differing perspectives in order to come to a more comprehensive understanding of what the fundamental solution may be<br><br>Kim – Archetypes I (2)<br>• Depends on perspective – which may help point us to a more fundamental solution<br><br>Kim – Archetypes II (3)<br>• Identify the original problem symptom<br>• Map the quick fixes (see fixes that fail)<br>• Identify impact on others<br>• Identify fundamental solutions<br>• Map the side effects – this is key because the side effects keep undermining the real solution in a shifting the burden<br>• Find interconnections to fundamental loops<br>• Identify high leverage actions – using a birds eye view can help show the gridlock situation – so that you're able to identify action.<br><br>Kim – Archetypes III (4)<br>• Break out of a 'reactive orientation' to a 'generative orientation'<br>• It might take more time, but it addresses the fundamental problem<br>• Generative orientation --> clarify of vision, clarity of core competencies<br><br>Meadows (5)<br>• "Breaking an addiction is painful. It may be the physical pain of heroin withdrawal, or the economic pain of a price increase to reduce oil consumption, or the consequences of a pest invasion while natural predator populations are restoring themselves. Withdrawal means finally confronting the real (and usually much deteriorated) state of the system and taking the actions that the addiction allowed one to put off. Sometimes the withdrawal can be done gradually. Sometimes a nonaddictive policy can be put in place first to restore the degraded system with a minimum of turbulence (group support to restore the self-image of the addict, home insulation and high-mileage cars to reduce oil expense, polyculture and crop rotation to reduce crop vulnerability to pests). Sometimes there's no way out but to go cold turkey and just bear the pain."

	• "The problem can be avoided up front by intervening in such a way as to strengthen the ability of the system to shoulder its own burdens. This option, helping the system to help itself, can be much cheaper and easier than taking over and running the system—something liberal politicians don't seem to understand."<br>• "Why are the natural correction mechanisms failing? • How can obstacles to their success be removed? • How can mechanisms for their success be made more effective?"<br>• "Again, the best way out of this trap is to avoid getting in. Beware of symptom-relieving or signal-denying policies or practices that don't really address the problem. Take the focus off short-term relief and put it on long-term restructuring."<br><br>Wolstenholme (6)<br>• See Figure B2.
Escalation	Kim – Archetype Basics (1)<br>• What is the relative measure that pits one party against another and what can you do to change it?<br>• What are significant delays int eh system that may distort the true nature of the threat?<br>• What are the deep-rooted assumptions that lie beneath the actions take in response to the threat?<br><br>Kim – Archetypes I (2)<br>• "The escalation archetype is about insecurity"<br>• "It can help to gain some perspective"<br>o Who are the parties whose actions are perceived as threats<br>o What is being threatened, and what is the source of that threat?<br>o What is the relative measure that pits one party against the other – and can you change it?<br>o What are the significant delays in the system that may distort the true nature of the threat?<br>o What are the deep-rooted assumptions that lie beneath the actions taken in response to the threat?<br><br>Kim – Archetypes II (3)<br>• Identify the competitive variable<br>• Name the key players<br>• Map what is being threatened (e.g. market share, or reputation?)<br>• Reevaluate relative measure – can the foundation be shifted so you're not really in the game?<br>• Quantify significant delays (e.g. short-term relief vs. long term investment)<br>• Identify larger goal<br>• Avoid the trap – easiest way is to not get in it in the first place.<br><br>Kim – Archetypes III (4)<br>• Best time to deal with it is early in the process (e.g. tit for tat stage rather than all-out)<br><br>Meadows (5)

	• "One way out of the escalation trap is unilateral disarmament—deliberately reducing your own system state to induce reductions in your final competitor's state. Within the logic of the system, this option is almost unthinkable. But it actually can work, if one does it with determination, and if one can survive the short-term advantage of the competitor."<br>• "The only other graceful way out of the escalation system is to negotiate a disarmament. That's a structural change, an exercise in system design. It creates a new set of balancing controlling loops to keep the competition in bounds (parental pressure to stop the kids' fight; regulations on the size and placement of advertisements; peace-keeping troops in violence-prone areas). Disarmament agreements in escalation systems are not usually easy to get, and are never very pleasing to the parties involved, but they are much better than staying in the race."<br>• "The best way out of this trap is to avoid getting in it. If caught in an escalating system, one can refuse to compete (unilaterally disarm), thereby interrupting the reinforcing loop. Or one can negotiate a new system with balancing loops to control the escalation."<br><br>Wolstenholme (6)<br>• See Figure B4.
Limits to success	Kim – Archetype Basics (1)<br>• The archetype is most helpful when it is used well in advance of any problems, to see how the cumulative effects of continued success might lead to future problems.<br>• Use the archetype to explore questions such as, "What kinds of pressures are building up in the organization as a result of the growth?"<br>• Look for ways to relieve pressures or remove limits before an organizational gasket blows.<br><br>Kim – Archetypes I (2)<br>• Same as Archetype basics<br><br>Kim – Archetypes II (3)<br>• Identify the growth engines – growth loops not just factors<br>• Identify potential limits and balancing loops<br>• Determine required change (to the limit)<br>• Assess time needed to change<br>• Balancing the growth – this has to been balanced with the capacity to adjust the limit as time goes on.<br>• Re-evaluate the growths strategy – it doesn't have to grow just for growth's sake.<br><br>Kim – Archetypes III (4)<br>• "As you plan for growth, automatically assume that something will eventually limit your expansion, and then look for those limits"<br>• "Does not lie in pushing harder on the engine of growth, but in finding and managing the factor or factors that are limiting success while you still have the time and money to do so."<br><br>Wolstenholme (6)

	• See Figure B1.
Accidental Adversaries	Kemeny (7)<br>• "Lack of coordinated action between two parties"<br>• "Strengthen your understanding of your partner's fundamental needs"

References

1. Kim DH, Anderson V. System Archetype Basics Waltham, Massachusetts: Pegasus Communications, Inc; 1998.
2. Kim DH. Systems Archetypes I: Diagnosing Systemic Issues and Designing High-Leverage Interventions. 2000.
3. Kim DH. Systems Archetypes II: Using Systems Archetypes to Take Effective Action 2000.
4. Kim DH. Systems Archetypes III: Understanding Patterns of Behavior and Delay. 2000.
5. Meadows DH. Thinking in systems: A primer: chelsea green publishing; 2008.
6. Wolstenholme E. Using generic system archetypes to support thinking and modelling. System Dynamics Review. 2004;20(4):341-56.
7. Kemeny J. "Accidental Adversaries": When Friends Become Foes. The Systems Thinker1994.
